# Disability and Fatigue in Multiple Sclerosis: Can Rehabilitation Improve Them through a Structured Retraining Program?

**DOI:** 10.1155/2022/7908340

**Published:** 2022-06-16

**Authors:** María José Arriaza, Azanzazu Vazquez, Teresa Hernández, David Varillas-Delgado, Virginia Meca-Lallana

**Affiliations:** ^1^Department of Rehabilitation and Physical Medicine, Hospital Universitario de La Princesa, 28062 Madrid, Spain; ^2^Autonomous University of Madrid, Madrid, Spain; ^3^Department of Neurorehabilitation, Hospital Universitario de La Princesa, 28062 Madrid, Spain; ^4^Department of Physiotherapy, Hospital de la Princesa, 28062 Madrid, Spain; ^5^School of Health Sciences, Francisco de Vitoria University, 28223 Pozuelo de Alarcón, Madrid, Spain; ^6^Department of Multiple Sclerosis, Hospital Universitario de La Princesa, 28062 Madrid, Spain

## Abstract

Functional rehabilitation programs in multiple sclerosis have demonstrated their efficacy in improving fatigue. The assessment of functional impairment, however, is more difficult. The purpose is to assess fatigue and disability as a first study measure and to verify their improvement after a specific functional rehabilitation program. An analytical, longitudinal, prospective, and experimental study was carried out with 51 patients aged 18-55 years, with an Expanded Disability Status Scale (EDSS) between 2 and 6.5 who were being followed up in outpatient clinics of the Rehabilitation Service of La Princesa Hospital. The fatigue and disability outcomes before and after a structured exercise training program were evaluated, with each subject acting as their own control. The variables were measured using the Modified Fatigue Impact Scale (MFIS), Barthel Index (BI), and Functional Independence Scale (FIM). Differences according to recurrent or progressive course of the disease are assessed. Improvement in the FIM scale was observed after the retraining program (*p* = 0.016) and was maintained in the medium term (*p* = 0.042). This improvement is not statistically significant in Barthel Index. Improvement in MFIS is observed after the program (*p* < 0.001) and 4-6 months after the end. Both disease courses experience the same improvements with no statistically significant differences between them. The retraining program improves fatigue and multiple sclerosis-related functionality in the short and medium term. There are no differences according to disease course. Both experience the same positive changes with our intervention.

## 1. Introduction

Multiple sclerosis (MS) is an immune-mediated, neurodegenerative disease with chronic inflammation of the central nervous system (CNS). It is characterized by inflammation, demyelination, and primary and secondary axonal injury [1–3]. It is the most common nontraumatic disabling neurological disorder in young adults and is one of the leading causes of disability in this age group [4–6].

The cause of MS is unknown but is known to involve genetic susceptibility and environmental factors. Because there is no known cure for MS, the main goals of treatment are to delay disease progression and improve patients' quality of life by treating MS-associated symptoms such as fatigue and functional deficits.

Fatigue is one of the most disabling manifestations of MS, a symptom that occurs in half of all patients [7–9]. Is a subjective manifestation of two components: a primary central component, based on dysfunction of the thalamic circuits, basal ganglia, and frontal cortex, and a secondary peripheral component, related to the rest of the manifestations of the disease (loss of motor capacity, spasticity, psychological disorders, sleep disturbances, etc.) [8].

Since the neurophysiological mechanisms are not exactly known, the treatment of fatigue in MS in clinical practice is challenging, combining nonpharmacological interventions with pharmacological treatments.

The assessment of functional defects associated with multiple sclerosis is very difficult, both in categorizing deficits in each patient and in assessing response to treatment. However, it is important in order to be able to compare the evolution of patients in an objective way and to be able to analyze the effects of treatments, including training programs.

As disability worsens, functional exercise capacity decreases in these patients [9].

In addition to existing pharmacological measures, nonpharmacological interventions include aerobic training, resistance training, and psychological and behavioral approaches, focusing on reducing symptoms, reducing exacerbations, slowing functional deterioration, and increasing exercise capacity. Treatment with functional rehabilitation programs has been used in recent years with high efficacy in MS patients. Several studies have proven the efficacy of adapted exercise in improving fatigue [10, 11].

In 85% of cases, MS starts as a relapsing-remitting (RR) disease in this form of the disease but can also be progressive in 15% of cases [12]. For this, the aim of this study is to analyze the efficacy of an educational and therapeutic program, based on aerobic retraining and targeted exercise instruction that is aimed at improving fatigue and disability and evaluate and compare the changes that occur after the functional rehabilitation program in patients with relapsing-remitting MS versus those with a progressive course.

## 2. Materials and Methods

### 2.1. Study Design and Sample

Longitudinal, prospective, experimental analytical study that evaluating the response of a group of patients with Expanded Disability Status Scale (EDDS) before and after a structured training program, each subject acting as their own control.

We calculated sample size to be 300 patients in the rehabilitation service by year, a single population proportion formula and based on 95% level of confidence, maximum variability of attributes with proportion of 0.5, plus or minus 15% points of relative error, a design effect of 1.2, and a nonresponse rate of 10%. According to the results provided by this estimate, fifty-one patients were included in the study. The baseline functional status was compared and the resulting short- and medium-term response to treatment (at the end of the program and at 4-6 months later), who met all the inclusion criteria, which were as follows:

- Patients with relapsing recurrent multiple sclerosis (RR), primary progressive (PP), or secondary progressive (SP)
- Ages between 18 and 55 years
- EDSS of 2 or more with at least a 3 in pyramidal functional system (FS) [13]
- Cognitive and training capacity to understand the treatment protocol correctly
- Acceptance and signature of the informed consent

Exclusion criteria are as follows:

- Presence of psychiatric diseases
- Life-threatening comorbidity in the short term (severe liver disease, cardiovascular disease, etc.)
- Osteoarticular disorders that prevent physical activity
- Active alcoholic habits
- Epilepsy
- Cognitive impairment
- Pregnancy or breastfeeding
- EDSS ≥ 6.5
- Having completed an exercise retraining program in the last year

All the patients agreed to participate in the study by signing informed consent. The study was evaluated and accepted by the Clinical Research Ethics Committee (CIEC) with Registration No: 2847, Approval (15-09-16) and is consistent with good clinical practice and regulatory requirements. The information provided by patients who freely and voluntarily agreed to participate in the study, and by their respective physicians, was used solely for the purposes of the study. The confidentiality of patients was followed in compliance with the Declaration of Helsinki 1964 (last update 2013).

### 2.2. Assessment Variables

- Sociodemographic data
- Assessment of the patient's global function (Barthel) and functional independence using the FIM scale
- Fatigue assessment before, after the program and 4-6 months after retraining using the MFIS scale

At the baseline visit, a complete physical examination will be carried out, as well as the patient's medical history. Data regarding their sociohealth situation will also be collected and a functional assessment will be carried out.

Clinical variables were analyzed before the program, after the program and 4-6 months later [14].

### 2.3. Associated Treatments

The pharmacological treatments of each patient are recorded at baseline and are not changed by entering the study. We better assess the results of the training program, and it better reflects the reality of patient care, as it is a study of routine clinical practice.

### 2.4. Clinical Intervention Protocol

After reviewing the existing literature, we designed an exercise retraining treatment program with two levels of aerobic exercise intensity. Inclusion of patients in the corresponding group was based on EDSS score [14].

The program was based on an exercise and aerobic endurance retraining protocol with a number of 8 alternating one-hour sessions (for three weeks two days a week, for two weeks one day a week). The program is supervised by the same physiotherapist in the same room and under the same conditions. Fatigue, dyspnea, temperature, humidity, heart rate, respiratory rate, and blood pressure were monitored during the sessions. The patient was instructed to be able to continue the protocol at home after the program (Table 1).

Each session started with stretching and muscle warm-up phase (functional exercises, mobility techniques with/without Kein ball). This was followed by aerobic work on a cycle ergometer (initial warm-up phase, work phase, and cool-down phase) lasting 30 minutes, where the maximum heart rate should not be exceeded. Analytical stretching, proprioceptive exercises, coordination, transfer and strengthening exercises were then performed until learning was complete [12].

Patients were instructed on the frequency and intensity of exercise and were provided with the appropriate documentation to be able to continue the work at the end of the 8 weeks, on their own, establishing frequency and duration.

### 2.5. Assessment of Effectiveness

Two widely used outcome measures to describe the level of independence in basic activities of daily living (ADLs) are the Functional Independence Measure (FIM) and the Barthel Index (BI). Both scales have a similar capacity to detect changes in disability in MS patients [15, 16].

The FIM is an objective and validated assessment of functional status that is widely used in rehabilitation clinics. Due to the fact that it allows direct observation of patients and that performance-based assessments are carried out by multidisciplinary teams—including physicians, therapists and nurses—the FIM is considered the gold standard test for functional assessments [17].

In the FIM scale [15, 16], 18 items on basic and instrumental activities of daily living are assessed (consisting of 6 items, which are subdivided into 4 motor and 2 cognitive items, which are further subdivided into 13 and 5 subareas, respectively). Ordinal scale of 18 ADLs measured from level 1 (total assistance) to level 7 (total independence).

Barthel Scale [16] measures the patient's global function. It includes a total of 10 items on basic activities of daily living. It is related to the patient's level of dependence.

Measurement of fatigue severity and its impact on daily life can be carried out by different scales. The most commonly used are the Fatigue Severity Scale (FSS) and the Modified Fatigue Impact Scale (MFIS). While the FSS can be answered very quickly (only 9 questions), the MFIS consists of 21 questions, which although it may be longer for routine clinical practice, can give a more accurate description of the impact of fatigue on the subject's daily activities [18].

Modified Fatigue Impact Scale (MFIS): MFIS [19] consists of 21 items that collect information on physical, cognitive, and psychosocial effort. Each item is scored from 0 to 4 points (0 = never; 1 = rarely; 2 = sometimes; often = 3; 4 = almost always) from 0 to 84 points. The higher level of MFIS generates the higher perception of fatigue.

The effectiveness of our program was also assessed by comparing the relapsing-remitting course with the progressive course of the disease.

### 2.6. Statistical Analysis

Results were analyzed using SPSS 21. Qualitative variables were presented as frequencies and percentages, while quantitative variables were presented as means and standard deviation when normally distributed and as median and interquartile range when not normally distributed. Variables were measured using the Modified Fatigue Impact Scale (MFIS) and the Barthel Index (BI) and Functional Independence Scale (FIM). Differences according to recurrent or progressive course of the disease are assessed.

For the inferential analysis of continuous variables, the Kolmogorov-Smirnov test was used to test for normality. In the case of normal distribution, parametric tests were used (Student's *t*-test, ANOVA), while in the case of nonnormality, nonparametric tests were used (Mann–Whitney *U*, Kruskal-Wallis). Dichotomous qualitative variables were analyzed by Chi-square test and contingency tables with Fisher adjustment. Statistically significant values were those with a value of *p* < 0.05.

## 3. Results and Discussion

51 patients with MS were included in the study, of whom the median age was 53 years, interquartile range 14 years [45–59]. Of the study patients, 33 patients had relapsing multiple sclerosis (64.7%), while 18 had progressive multiple sclerosis (35.3%).

The most frequent EDSS score was 6.0 (29.4%), (needing some support to walk 100 meters, with or without rest) and score 3.0 (19.6%), moderate disability in one functional system, or mild disability in three or four functional systems in the absence of walking difficulty. The remaining patients had lower scores.

Homogeneity was demonstrated between the groups of patients in the study, recurrent course and progressive course in the variables at the beginning of the study, which validates the comparison that we are going to carry out between the different scales in the study.

**Figure 1:**
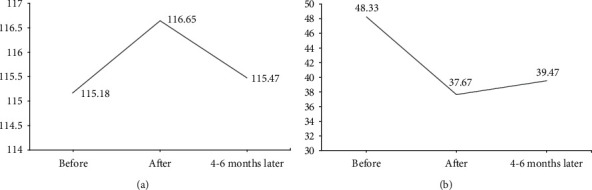
(a) Functional independence scale (FIM) data at follow-up measurements in study participants. (b) MFIS scale data on follow-up measurements in study participants.

Following Figure 1(a), Functional Independence Scale (FIM) obtained a mean value of 115.18 (±9.693), at immediate posttreatment, a mean value of 116.65 (±8.353), and at 4-6 months, a value of 115.47 (±8.319). Barthel scale was presented in median and interquartile range, being at the beginning median value 90 and interquartile range 80-100, after the program median value 95 and interquartile range 85-100; and at 4-6 months later median value 90, with interquartile range 80-100. MFIS scale mean value before program was 48.33 (±18.636), at after 37.67 (±19.547), and at 4-6 months later, the mean values corresponded to 39.47 (±18.119) (Figure 1(b)).

FIM showed statistical differences between baseline and postintervention (*p* = 0.016) and at 4-6 months later compared to after program (*p* = 0.042). For the Barthel Index score, in the follow-up periods, there were no differences. MFIS showed statistical differences were observed between before the program values versus after treatment (*p* < 0.001), as well as values between before and 4-6 months later (*p* < 0.001), reporting no differences in the medium term (*p* = 0.783) (Table 2).

The results of FIM, between progressive (P) and relapsing-remittent (RR) groups, scores were analyzed in the groups before treatment, after treatment and at 4-6 months, with no statistical differences between the groups after treatment (*p* = 0.208) or later (*p* = 0.197). Barthel Index was compared, with no statistical differences between groups at baseline (*p* = 0.242), posttreatment (*p* = 0.474), and 4-6 months later (*p* = 0.765) in the measured scores. Finally, MFIS assessment was analyzed in the groups at baseline (*p* = 0.721), after treatment (*p* = 0.641), and 4-6 months later (*p* = 0.944), with no statistical differences between groups (Table 3).

## 4. Discussion

MS is a disabling disease, given its chronic neurodegenerative course [6]. It affects a young population with significant functional requirements. MS can lead to long-term physical and mental impairment affecting basic activities of daily living. In an attempt to minimize the disability associated with MS, rehabilitation plays a key role [20].

Among the many rehabilitation interventions that can be implemented to maintain patients' functionality is the development of health education programs that include disease-related exercise guidelines [10–12, 20].

Given the available health resources, the multidisciplinary rehabilitation approach may be a nonpharmacological and specific treatment alternative to slow functional disability and, therefore, the implementation of rehabilitation programs would contribute to the physical and mental well-being of patients with this disease. The program's development was based on the existing evidence and the duration was based on effort retraining programs that have been shown to be effective in other pathologies.

Latimer-Cheung et al. in their article published in 2013 should be 30 minutes of moderate-intensity aerobic exercise and strength training, performed twice a week to achieve benefits. They also underline the importance of supervised exercise. Dalgas et al. recommend a longer time interval of 10-40 minutes of moderate to vigorous exercise 2-3 times per week [21]. Evidence-based clinical practice guidelines and physical activity guidelines for MS patients have been developed according to international standards [11]. Latimer-Cheung et al. conducted a systematic review of the evidence on the effects of physical training on fitness, mobility, and fatigue in MS patients. They concluded that among people with mild to moderate disability, there is sufficient evidence to conclude that training is effective in improving aerobic capacity and muscle strength and may also improve mobility, fatigue, and quality of life [10]. A Cochrane review by Rietberg et al. in 2005 concluded that there is level 1 evidence in favor of the benefit of exercise in terms of muscle power, adaptation to exertion, and improvement of motor activities [20].

### 4.1. Fatigue

Treatment through functional rehabilitation programs has been used in recent years with high efficacy in MS patients. Several studies have evaluated the efficacy of adapted exercise in improving fatigue [10, 11].

In 1992, the results of a trial were published involving 54 MS patients, who were randomly divided into two groups: exercise versus nonexercise. The intervention took 15 weeks, and the exercise group improved in almost all clinical scores and biomarkers [22].

Another more recent trial showed beneficial effects of aerobic exercise compared to placebo on MS fatigue. In particular, this trial compared exercise led by a physiotherapist, exercise led by a fitness instructor, yoga and placebo (no particular intervention) and showed that all three interventions were effective (compared to placebo) in improving fatigue. In addition, the two exercise interventions also showed an effect on scores related to objective physical disability [23].

Exercise therapy could induce a positive effect on fatigue, but the results are heterogeneous because the sample of many studies is of patients with low fatigue. In addition, there are few studies that have assessed fatigue as a first study measure [19].

A Cochrane systematic review conducted in 2015 has concluded that there is a significant effect of resistance and mixed exercise in reducing fatigue in MS patients [14], but in most of the assessed studies, the measurement scales are not validated for fatigue and patient characteristics were not explicitly detailed [12].

As for nonpharmacological approaches, they can be broadly divided into physical, psychological, and mixed physical/psychological interventions. Several studies, many of them randomized clinical trials, support the use of all these types of nonpharmacological interventions to treat MS-related fatigue.

Recent publications suggest that the application of mixed approaches can have excellent results in clinical practice, not only in relation to fatigue levels but also to more general aspects of MS. This training program demonstrates benefits on fatigue in patients with long-standing MS as measured by the MFIS scale. It is currently unclear what constitutes a change in fatigue in people with MS. This restricts the interpretation of treatment efficacy when trying to evaluate fatigue interventions [24].

If we compare both disease courses, there is no difference between them; regardless of whether they present in a progressive or relapsing form, the improvement in fatigue after the program is visible in all of them. According to the study by Rooney et.al, it is considered a significant change in fatigue if the difference in the MFIS is 4 points [24]. Our patients have exceeded this score, so we can affirm that the improvement in fatigue has a significant effect on the quality of life of these patients. However, fatigue cannot be addressed with multimodal rehabilitation alone, as pharmacological therapy can play a key role in alleviating this symptomatology.

### 4.2. Disability

The importance of assessing disability outcomes is well recognized in MS patients. The FIM was developed to be a more comprehensive measure of disability than the BI. Although the FIM is widely used and has proven to be reliable and valid, information on its ability to be responsive is limited, especially in comparison to the BI. Both disability measures are suitable for screening people with MS [15]. Freeman et al. carried out a study in a sample of 50 MS patients in which he found that the disability benefits were sustained for approximately 6 months, while the improvement in quality of life was sustained for 10 months [14].

The results of this study suggest that FIM has advantages over BI in assessing changes in disability due to therapeutic interventions, and like Freeman's study, the disability benefits are sustained over time. Both scales are widely used in clinical practice, but as other studies, the responsiveness of BI may be limited in the research context. There is a slight ceiling and floor effect more evident in the BI. This ceiling effect may explain why we did not find statistically significant differences with the BI; however, they appear on the FIM scale [16].

FIM is the gold standard test for functional assessments [17] and is the one that reports significant improvement after the program. There is a clear need for well-developed clinical research trials examining the use of FIM scores to predict functional outcomes [25].

The main limitations of our study lie in the small sample size and limited follow-up. The program is structured in such a way that follow-up is carried out up to the first six months, with no evidence of maintenance of improvement at one year or 18 months. As it is standard clinical practice, all patients who meet the inclusion criteria and none of the exclusion criteria are included in the retraining.

## 5. Conclusions

The exercise training program improves multiple sclerosis-related fatigue in the short and medium term. It can be an effective adjunct to fatigue management in the absence of specific pharmacological treatment to improve it.

The program improves patient's functionality in the short and medium term. The FIM is completer and more sensitive than the Barthel Index. There are no important differences in functionality or fatigue according to the course of the disease. Both experience the same positive changes with our intervention.

## Figures and Tables

**Table 1:** Retraining program.

Retraining program	
(i) Energy-saving techniques	Information about your disease and symptoms, the factors that worsen it, and the factors that favor the onset of symptoms.
(ii) Transfer training and postural hygiene.	
(iii) Reflex inhibition and relaxation postures	Decubitus, sitting, and bipedestation.
(iv) Stretching and exercises for spasticity control	Insisting on functional exercises for triple flexion (decubitus and upright) osteoarticular alterations that prevent physical activity.
(v) Respiratory physiotherapy techniques	Passive, assisted, active mobilization
(vi) Mobility techniques and general active exercises.	
(vii) Neuromeningeal mobility techniques and proprioceptive neuromuscular facilitation techniques	
(viii) Frenkel exercises and proprioception exercises.	Coordination and functional balance (quadrupedal, seated, and standing).
(ix) Aerobic training on a cycleergometer or pedalier	According to the patient's functional situation. Classic endurance training for 30 minutes, at a constant power corresponding to the ventilatory threshold (UV1).
(x) Walking rehabilitation and stair training	

Source: own elaboration.

**Table 2:** Differences in FIM, Barthel Index, and MFIS scores in multiple sclerosis participants.

Period	MS (*n* = 51)	*p* value
FIM score		
Before, mean (SD)	115.22 (9.856)	0.016
After, mean (SD)	116.65 (8.353)	
After, mean (SD)	116.65 (8.353)	0.042
4-6 months later, mean (SD)	115.47 (8.319)	
Barthel index score		
Before, mean (SD)	87.96 (11.030)	0.372
After, mean (SD)	90.10 (10.532)	
4-6 months later, mean (SD)	87.65 (11.416)	
MFIS score		
Before, mean (SD)	48.37 (18.558)	<0.001
After, mean (SD)	37.67 (19.548)	
4-6 months later, mean (SD)	39.47 (18.119)	

SD: standard deviation; MS: multiple sclerosis.

**Table 3:** Differences in FIM score, Barthel Index, and MFIS scores between progressive and relapsing-remittent patients.

Period	P (*n* = 18)	RR (*n* = 33)	*p* value
FIM score			
Before, mean (SD)	113.00 (9.732)	116.36 (9.611)	0.240
After, mean (SD)	114.67 (6.589)	117.81 (9.127)	0.208
4-6 months later, mean (SD)	113.44 (8.979)	116.65 (7.821)	0.197
Barthel index score			
Before, mean (SD)	52.50 (17.092)	46.06 (19.297)	0.242
After, mean (SD)	40.33 (17.269)	36.13 (20.874)	0.474
4-6 months later, mean (SD)	40.50 (16.738)	38.87 (19.118)	0.765
MFIS score			
Before, mean (SD)	88.06 (8.599)	86.82 (13.099)	0.721
After, mean (SD)	89.17 (9.587)	90.65 (11.161)	0.641
4-6 months later, mean (SD)	87.50 (9.587)	87.84 (12.506)	0.944

SD: standard deviation; P: progressive; RR: relapsing-remittent.

## Data Availability

The database used for this study is property of La Princesa Hospital. The data is confidential information of patients that by the Data Protection Law and for the privacy of the patient as well as for ethical reasons I cannot provide. We do not have permission to link this data.
